# Optimal Breast Density Characterization Using a Three-Dimensional Automated Breast Densitometry System

**DOI:** 10.3390/curroncol28060448

**Published:** 2021-12-14

**Authors:** Reika Yoshida, Takenori Yamauchi, Sadako Akashi-Tanaka, Misaki Matsuyanagi, Kanae Taruno, Terumasa Sawada, Akatsuki Kokaze, Seigo Nakamura

**Affiliations:** 1 Department of Breast Surgical Oncology, Showa University School of Medicine, 1-5-8 Hatanodai, Shinagawa-ku, Tokyo 142-8555, Japan; sakashi@med.showa-u.ac.jp (S.A.-T.); mtyng@med.showa-u.ac.jp (M.M.); ktaruno@med.showa-u.ac.jp (K.T.); terusan@med.showa-u.ac.jp (T.S.); seigonak@gmail.com (S.N.); 2 Department of Hygiene, Public Health and Preventive Medicine, Showa University School of Medicine, 1-5-8 Hatanodai, Shinagawa-ku, Tokyo 142-8555, Japan; yamauchi-t@med.showa-u.ac.jp (T.Y.); akokaze@med.showa-u.ac.jp (A.K.)

**Keywords:** Volpara, breast density, breast neoplasms, mammography, ultrasonography

## Abstract

Dense breasts are a risk factor for breast cancer. Assessment of breast density is important and radiologist-dependent. We objectively measured mammographic density using the three-dimensional automatic mammographic density measurement device Volpara™ and examined the criteria for combined use of ultrasonography (US). Of 1227 patients who underwent primary breast cancer surgery between January 2019 and April 2021 at our hospital, 441 were included. A case series study was conducted based on patient age, diagnostic accuracy, effects of mammography (MMG) combined with US, size of invasion, and calcifications. The mean density of both breasts according to the Volpara Density Grade (VDG) was 0–3.4% in 2 patients, 3.5–7.4% in 55 patients, 7.5–15.4% in 173 patients, and ≥15.5% in 211 patients. Breast density tended to be higher in younger patients. Diagnostic accuracy of MMG tended to decrease with increasing breast density. US detection rates were not associated with VDG on MMG and were favorable at all densities. The risk of a non-detected result was high in patients without malignant suspicious calcifications. Supplementary use of US for patients without suspicious calcifications on MMG and high breast density, particularly ≥25.5%, could improve the breast cancer detection rate.

## 1. Introduction

Merely repeating mammography (MMG) examination in women with dense breasts may not reveal abnormal findings, resulting in a delayed breast cancer diagnosis, which has become a global issue [1,2,3,4,5,6,7,8]. Despite reports suggesting that the combined use of ultrasonography (US) and MMG is effective in detecting breast cancer [8,9,10], performing US for a vast number of individuals warranting screening is impossible.

Dense breasts are considered a strong risk factor for breast cancer. Statistical surveys on differences in mammographic density distribution due to racial differences indicated a higher frequency of denser breasts in Asians than in Westerners [1,11,12,13,14]. Even among Asians, mammographic density tends to be higher among Chinese than among Malay and Indian women [11] and is higher among Japanese women than among European and American women [15].

Previous reports indicate that a higher the breast density is associated with a higher incidence of breast cancer among Japanese women. However, although one of the causes of high breast density among Asians is a low BMI, and the cause of high breast density is different from that of Europeans, the incidence of breast cancer between Asians and Europeans is not appreciably different [16,17].

Mammographic density on MMG is classified into four categories according to the Breast Imaging-Reporting and Data System proposed by the American College of Radiology (ACR BI-RADS) as a global standard [18]. Nonetheless, this assessment depends on visual examination by a radiologist; thus, interpretations vary among clinicians [19]. A previous report demonstrated that for MMG screening tests performed at different times, 20% of women were classified by the same radiologist into different BI-RADS density categories [20]. Additionally, no clear criteria for the combined use of US and MMG have been proposed.

Several methods for the objective calculation of mammographic density from electronic MMG data have been investigated [21,22,23,24,25]. Volpara™ (Volpara Health Technologies, Wellington, New Zealand) is a three-dimensional automated breast densitometry system with unified assessment criteria for mammographic density. A significant positive correlation between breast densities determined by digital MMG software, such as Volpara, and those determined by radiologists has been reported [21,22,23,24,25].

Considering this background, we objectively measured mammographic density in Japanese women using the computer-based mammographic density measurement software Volpara and examined the diagnostic accuracy according to Volpara results. We examined objective and specific selection criteria for patients who should be screened with US in addition to MMG to improve the effective diagnostic rate in breast cancer screening.

## 2. Materials and Methods

### 2.1. Study Design

Patients who underwent breast cancer surgery between January 2019 and April 2021 in our hospital were reviewed. The exclusion criteria were as follows: (1) patients without MMG images obtained in our hospital, (2) patients whose tumors were breast cancer recurrences following partial mammectomy, and (3) patients with occult breast cancer and Paget’s disease. Patients with bilateral breast cancer were regarded as two independent cases. We conducted a case series study based on patients’ age, diagnostic accuracy, diagnostic category by MMG (with ≥3 being malignant), effects of detection by the combined use of US and MMG, pathological classification, preoperative hormone therapy/neoadjuvant chemotherapy, size of invasion, and presence of calcifications. The study was conducted according to the guidelines of the Declaration of Helsinki and was approved by the Showa University Institutional Review Board (No. 3371). Because all participants in this study were patients with breast cancer, patients whose condition was deemed malignant or benign by both MMG and US were considered as “detected” and “non-detected”, respectively.

### 2.2. Study Content and Investigated Items 

In our hospital, Senographe Essential (GE Medical Systems, Chicago, IL, USA) is used for MMG, LOGIQ E9 (GE Medical Systems) is utilized for US, and Volpara version 1.5.3 (Volpara Health Technologies) is used as the three-dimensional automatic mammographic density measurement device.

Volpara uses digital MMG raw data to perform a three-dimensional analysis of the breasts to calculate the volume and density of fat and mammographic density in each breast. Mammographic density is defined as the ratio (%) of the mammographic density volume divided by the entire breast volume, which is an objective and reproducible result using the average density of both breasts. The measured density was adapted to the four ACR BI-RADS categories (A: fatty; B: scattered; C: heterogeneously dense; and D: extremely dense) [16], which are widely used worldwide, and was shown in terms of Volpara Density Grade (VDG): a–d. Furthermore, the average density in both breasts was defined as follows: 0–3.4%, VDG: a; 3.5–7.4%, VDG: b; 7.5–15.4%, VDG: c; and ≥15.5%, VDG: d.

### 2.3. Statistical Analysis

Patients’ age, breast density (%) as measured by Volpara, and size of invasion were assessed as continuous variables. Given that as a normal distribution could not be assumed, between-group differences were tested using the Mann–Whitney U test, whereas significant differences between multiple groups were examined using the Kruskal–Wallis test. Multiple comparisons were performed using the pairwise Wilcoxon test and *p*-value correction with Holm’s method. The degree of correlation was assessed based on the Spearman’s rank correlation coefficient. Furthermore, the presence of calcifications, VDG, presence of invasion, diagnosis by MMG, and diagnosis by US were assessed as categorical variables, and the relationship between categorical variables was assessed using Fisher’s exact test. The Cochran–Armitage test was used for trend analysis. Confounding factors were adjusted and analyzed using logistic regression analysis for diagnostic accuracy and non-detected rate.

As the size of invasion was determined using the pathological specimen after surgery, it was examined in 385 patients, except in patients who received neoadjuvant chemotherapy or preoperative hormone therapy and patients for whom all lesions had been removed by biopsy. 

## 3. Results

Of the 1227 patients reviewed, 441 met the inclusion criteria and were included for further analyses. Table 1 presents the breakdown of background data for these 441 patients. The median age was 56 years, the median Volpara density was 14.7%, and the median size of invasion was 12.5 mm. Of all patients, 81.4% and 89.1% were categorized as “detected” by MMG and US, respectively.

### 3.1. Volpara Density by Age and Relationship between VDG and Age

**Figure 1:**
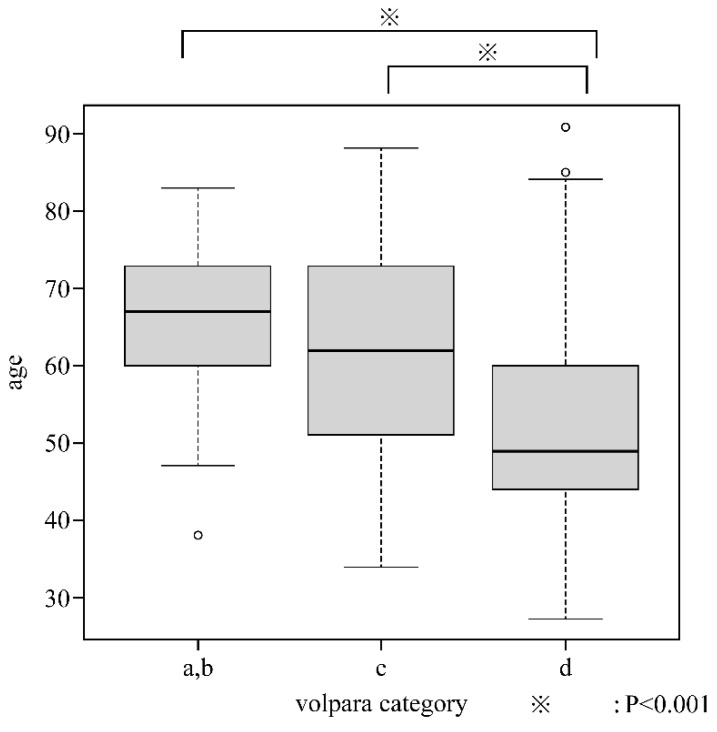
Relationship between Volpara Density Grade and age.

A negative correlation between age and VDG was observed (*p* = −0.47, *p* < 0.001). Patients were classified based on VDG as follows: VDG: a, 2 (0.5%); VDG: b, 55 (12.5%); VDG: c, 173 (39.2); and VDG: d, 211 (47.8%). As there were two patients in category a, subsequent analyses were conducted by including them in VDG: b, such that there were 57, 173, and 211 patients in VDG: a + b, c, and d, respectively. We identified no significant difference in age between VDG: a, b, and c, but significant differences were observed between VDG: a, b, and d as well as between c and d (Figure 1).

**Figure 2:**
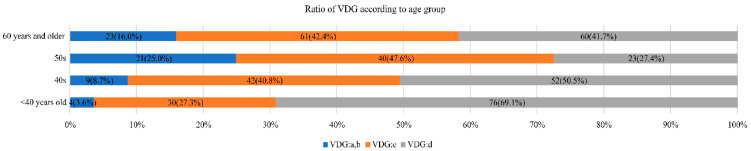
Volpara Density Grade (VDG) according to age.

Figure 2 shows the proportion of VDG according to age. VDG: d tended to be more common at younger ages (*p* < 0.001). The proportion of VDG: c and d, which would be regarded as dense breasts, accounted for 96.3% (106/110) of patients aged <40 years, 91.2% (94/103) of patients in their 40s, 75% (63/84) of patients in their 50s, and 84.7% (121/144) of patients aged ≥60 years.

### 3.2. Relationship between VDG and Diagnostic Accuracy

The diagnostic accuracy of MMG was 81.4% (359/441) among all patients (84.2% [48/57], 84.2% [148/173], and 77.3% [163/211] for patients in VDG: a + b, c, and d, respectively), highlighting a significant trend in which the diagnostic accuracy decreased with increasing VDG (*p* = 0.0284).

The diagnostic accuracy of US was 89.1% (393/441) among all patients (87.7% [40/57], 89.6% [155/173], and 89.1% [188/211] for patients in VDG: a + b, c, and d, respectively), indicating no difference in the diagnostic accuracy among VDG categories.

Ten patients in our study had non-detected conditions by both MMG and US. One of these ten patients was revealed to have cancer, as shown by biopsy conducted as a precaution because of a mild increase in calcifications. Another patient being monitored for a fibroadenoma-like mass by follow-up US was also diagnosed with cancer, as revealed by a biopsy performed due to a mild increase in the tumor size. The cancers on the contralateral breast in the remaining eight patients were accidentally determined by preoperative magnetic resonance imaging (MRI).

### 3.3. Diagnostic Accuracy by US for Patients Whose Tumors Were Non-Detected by MMG According to VDG

The diagnostic accuracy of US among patients in whom breast cancer was non-detected by MMG was 87.8% for all such patients, 88.9% in VDG: a + b, 76.0% in VDG: c, and 93.8% in VDG: d. No correlation with VDG was observed. 

### 3.4. Impact of VDG on the Relationship between Non-Detected Cases by MMG and Presence of Invasion

**Figure 3:**
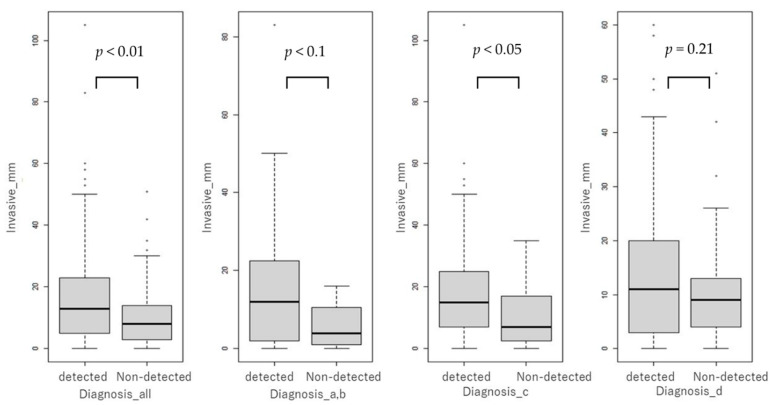
Size of invasion in the non-detected and detected groups, as shown by mammography.

The diagnostic accuracy of MMG was not significantly associated with the presence of invasion in all VDG groups (*p* > 0.05). The comparison of the size of invasion between the non-detected and detected groups indicated that the size of invasion was significantly smaller in the non-detected group than in the detected group in both complete patient populations (*p* < 0.01) and the VDG: c group (*p* < 0.05). A nonsignificant trend for a smaller size of invasion was observed in the VDG: a + b group (*p* < 0.1), and no significant difference was noted in the VDG: d group (*p* = 0.21) (Figure 3).

### 3.5. Difference in Non-Detected Rate between Cases with and without Calcifications Requiring MMG Differentiation by VDG

Of 192 patients with malignant suspicious calcifications (suspicious calcifications), 5.2% (10/192) were non-detected on MMG, whereas 28.9% (72/249) of 249 patients without calcifications had non-detected results on MMG (Table 2). The risk of a non-detected result was 7.4 times greater among patients without suspicious calcifications (*p* < 0.001).

**Figure 4:**
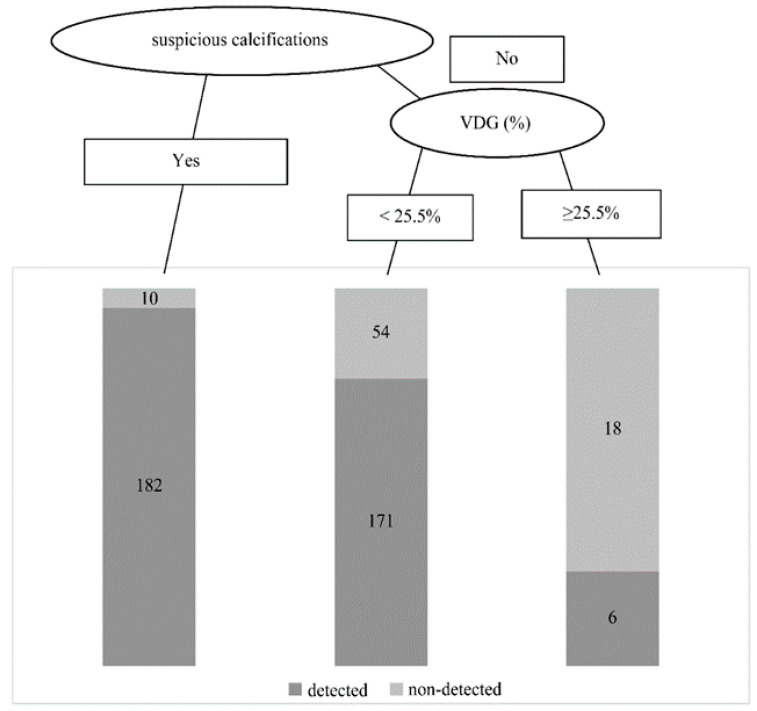
Risk factors for non-detected results and flow diagram leading to a non-detected result. VDG—Volpara Density Grade.

The MMG results of all patients were categorized into four groups (“MMG correct diagnosis group with suspicious calcifications”, “MMG non-detected group with suspicious calcifications”, “MMG correct diagnosis group without suspicious calcifications”, and “MMG non-detected group without suspicious calcifications”), and their VDG results were compared. This comparison indicated that patients in the “MMG non-detected group without suspicious calcifications” and “MMG correct diagnosis group without suspicious calcifications” had a significantly higher mammographic density (Figure 4).

Logistic regression analysis of age, VDG, and suspicious calcifications was conducted for non-detected patients, revealing that VDG and the presence of suspicious calcifications were significant risk factors for a non-detected result. The risk of a non-detected result was 1.07 times greater when the VDG increased by 1%, increasing to 9.2 times when calcifications showed no malignant findings (Table 3). In particular, the non-detected rate increased sharply when VDG was ≥25.5% (Figure 4).

## 4. Discussion

The results of our study suggest that the combined use of US would be beneficial in patients without suspicious calcifications by MMG and classified as Volpara density of VDG: d or higher (in particular, those ≥ 25.5%). When these classifications were applied to the present results, the combined use of US increased the detection sensitivity to 95.8% (23/24) from 25% (6/24) with MMG alone.

As mentioned earlier, merely repeating MMG examinations in women with dense breasts can lead to masking and oversight of abnormal findings, resulting in a delay in breast cancer diagnosis [1,2,3,4,5,6,7]. Several studies using various supplemental modalities have been conducted to resolve this problem.

In the United States, breast cancer screening using MMG for women with fatty breasts showed a sensitivity of 80%; nonetheless, the diagnostic accuracy significantly decreased to 30% in women with extremely dense breasts [26]. As such, testing with MMG alone has been suggested to be insufficient for dense breasts. Thus far, the combined use of US and MMG has been proposed to overcome this shortcoming. Previous reports indicated that 89% of patients diagnosed with invasive breast cancer tested positive on MMG. However, the rate of detection increased to 98% through the combined use of US [27], the rate of detection of breast masses with a small size of invasion also increased through the combined use of US and MMG [28]. Invasive breast cancer can be detected early by using both strategies for screening women with dense breasts [29]. Furthermore, the sensitivity was significantly higher when both MMG and US were used for women with dense breasts (77.5%) than when MMG alone was used (50%) [30]. The J-START, a large randomized controlled trial conducted in Japan to examine the usefulness of adding US to MMG examinations for women in their 40s with a high prevalence of dense breasts [31,32], demonstrated that the combined use of US and MMG increased the rate of breast cancer detection by 1.5-fold, as compared with the use of MMG alone. Furthermore, the combined use of US resulted in increased sensitivity, regardless of breast density, indicating that combined US is the best method for these women. In addition, for patients with suspicious calcification and already considered malignancy, combined US is very helpful in determining abnormal findings in other sites, enlargement of axillary lymph nodes, invasion of the pectoralis major muscle, and treatment strategy. However, combined use of US in all cases is difficult due to a shortage of human resources, increases in false positive case, and poor cost-effectiveness.

Recently, reports have demonstrated the utility of combined use of various supplemental modalities such as three-dimensional automated breast ultrasound (ABUS), MRI, and digital breast tomosynthesis (DBT) for MMG screening.

ABUS is a highly reproducible and operator-independent imaging method. The addition of ABUS to MMG screening increased the sensitivity of detecting invasive ductal carcinomas in dense breasts by approximately 40% [33,34]. ABUS includes computer-aided detection as auxiliary software, which can reduce the reading time without compromising the screening accuracy [35,36]. A study conducted in 2021 compared screening with MMG alone and with ABUS in 1165 women with dense breasts and reported that ABUS combined with MMG increased the sensitivity by 31.8% [37]. Future challenges include further examination of cost-effectiveness and, as with portable US (i.e., handheld US), reducing the risk of false positives [38].

In recent years, contrast-enhanced spectral MMG has also been performed. There is also the accelerating research field of radiomics, which seeks to integrate and combine information from multiple imaging diagnoses, such as CT, MRI, and US, and analyze it using AI to improve the rate of correct diagnosis. This has the potential to improve diagnostic performance even in dense breasts [39].

The DENSE trial, a randomized controlled trial of MRI screening in the Netherlands, compared the incidence of interval cancers over 2 years in 40,373 women between 50 and 75 years with normal MMG screening results and VDG: d breast density. Participants were divided into two groups: those who underwent screening with MMG only and those who underwent screening with both MRI and MMG. The results revealed that cancer was detected at an earlier stage in the group with combined MRI and MMG. However, the biggest drawback of this test was that it requires the use of contrast media [40]. Although MRI has an extremely high cancer detection rate, it is very time-consuming and expensive for screening. In contrast, AB-MRI is less expensive, time-consuming, and less complicated than MRI, while maintaining the same diagnostic performance [36,41]. The use of DBT in combination with MMG screening for invasive cancer significantly improves both the sensitivity and specificity of detection [42,43,44]. Nonetheless, Lowry et al. reported that women with extremely dense breasts did not benefit appreciably from the addition of DBT [45]. Studies comparing DBT and AB-MRI with respect to cost-effectiveness have reported that AB-MRI is recommended if the cost is below a certain amount [46]. The eight cases in which preoperative MRI incidentally revealed contralateral breast cancer may indicate the potential usefulness of this AB-MRI.

As mentioned above, the optimal modality for breast cancer screening remains a topic of discussion. Most previous studies on dense breasts were based on visual judgement rather than objective values acquired by automated measurements. Therefore, the results of this study, for which specific numerical values were used as the prognostic criteria, are considered objective and meaningful. 

Previous reports indicated that an increase in mammographic density reduces MMG sensitivity in patients without suspicious calcifications [10,47]. In another report, the addition of DBT for dense breasts did not improve early breast cancer detection without calcifications [48]. However, there are only a few studies from which conclusions can be drawn. According to the present study results, the risk of a non-detected result was 7.4-fold greater in patients without suspicious calcifications.

This study has some limitations. Given that this was a case series study, VDG (≥25.5%) should be confirmed by validation studies from actual clinical practice. Furthermore, because this study examined patients diagnosed with cancer, it was impossible to examine false negatives among patients who were not diagnosed with cancer or to examine interval cancer. In addition, there was an imbalance in sample sizes among the four VDG classifications, with only 2 out of 441 patients belonging to the group VDG: a, which is rare among Japanese women [15]. The widespread use of Volpara, other breast densitometry software and large-scale analyses at multiple centers [15] could enable a more accurate analysis of mammographic density according to ethnicity.

## 5. Conclusions

Active use of US in combination with MMG for patients without suspicious calcifications on MMG and a Volpara density classification of VDG: d (particularly for those ≥25.5%) could help improve the rate of effective breast cancer diagnosis. For such women, supplemental US is an efficient choice in breast cancer screening. A larger study is needed to validate these findings.

## Figures and Tables

**Table 1:** Breakdown of the patient population (*N* = 441).

Characteristic		Value
Age		56 [47, 70]
Volpara density		14.7% [9.2, 20.7]
Suspicious calcifications	Yes	193 (43.8%)
	No	248 (56.2%)
MMG category	1.2 (non-detected)	82 (18.6%)
	3.4.5 (detected)	359 (81.4%)
US category	1.2 (non-detected)	48 (10.9%)
	3.4.5 (detected)	393 (89.1%)
Neoadjuvant chemotherapy/preoperative hormone therapy	Yes	55 (12.5%)
	No	386 (87.5%)
Invasion	Yes	354 (80.3%)
	No	87 (19.7%)
Size of invasion		12.5 mm [5, 22]
Method of diagnosis	Symptomatic	81 (18.4%)
	Screening	147 (33.3%)
	Referred	129 (29.3%)
	Others	84 (19%)

MMG, mammography; US, ultrasonography. Presented statistics: Median [25%, 75%]; *n* (%).

**Table 2:** Non-detected rate by MMG in patients with and without suspicious calcifications.

Characteristics	Diagnosis by MMG Only		
Suspicious calcifications		Detected	Non-detected
	Yes	182	10 (5.2%)
	No	177	72 (28.9%)

MMG, mammography; *p* < 0.001.

**Table 3:** Risk factors for non-detected results by MMG (logistic regression analysis).

Characteristics		Odds Ratio	95% Confidence Interval	*p*
Age		0.99	0.97–1.01	0.3
VDG		1.07	1.04–1.11	<0.001
Suspicious calcifications	Yes	1.00 (reference)		
	No	9.2	4.68–19.85	<0.001

VDG—Volpara Density Grade.

## Data Availability

The data presented in this study are available on request from the corresponding author.

## References

[1] Boyd N.F., Rommens J.M., Vogt K., Lee V., Hopper J.L., Yaffe M.J., Paterson A.D. (2005). Mammographic breast density as an intermediate phenotype for breast cancer. Lancet Oncol..

[2] Harvey J.A., Bovbjerg V.E. (2004). Quantitative assessment of mammographic breast density: Relationship with breast cancer risk. Radiology.

[3] Jeffers A.M., Sieh W., Lipson J.A., Rothstein J.H., McGuire V., Whittemore A.S., Rubin D.L. (2017). Breast cancer risk and mammographic density assessed with semiautomated and fully automated methods and BI-RADS. Radiology.

[4] Kerlikowske K., Zhu W., Tosteson A.N., Sprague B.L., Tice J.A., Lehman C.D., Miglioretti D.L. (2015). Identifying women with dense breasts at high risk for interval cancer: A cohort study. Ann. Intern. Med..

[5] McCormack V.A., dos Santos Silva I. (2006). Breast density and parenchymal patterns as markers of breast cancer risk: A meta-analysis. Cancer Epidemiol. Biomark. Prev..

[6] Byrne C., Schairer C., Wolfe J., Parekh N., Salane M., Brinton L.A., Hoover R., Haile R. (1995). Mammographic features and breast cancer risk: Effects with time, age, and menopause status. J. Natl. Cancer Inst..

[7] Brentnall A.R., Cuzick J., Buist D.S.M., Bowles E.J.A. (2018). Long-term accuracy of breast cancer risk assessment combining classic risk factors and breast density. JAMA Oncol..

[8] Uematsu T., Nakashima K., Kikuchi M., Kubota K., Suzuki A., Nakano S., Iwata H. (2020). The Japanese Breast Cancer Society clinical practice guidelines for breast cancer screening and diagnosis. Breast Cancer.

[9] Zonderland H.M., Coerkamp E.G., Hermans J., van de Vijver M.J., van Voorthuisen A.E. (1999). Diagnosis of breast cancer: Contribution of US as an adjunct to mammography. Radiology.

[10] Kolb T.M., Lichy J., Newhouse J.H. (2002). Comparison of the performance of screening mammography, physical examination, and breast US and evaluation of factors that influence them: An analysis of 27,825 patient evaluations. Radiology.

[11] Mariapun S., Li J., Yip C.H., Taib N.A., Teo S.H. (2015). Ethnic differences in mammographic densities: An Asian cross-sectional study. PLoS ONE.

[12] Rajaram N., Mariapun S., Eriksson M., Tapia J., Kwan P.Y., Ho W.K., Harun F., Rahmat K., Czene K., Taib N.A.M. (2017). Differences in mammographic density between Asian and Caucasian populations: A comparative analysis. Breast Cancer Res. Treat.

[13] van Gils C.H., Otten J.D., Verbeek A.L., Hendriks J.H. (1995). Short communication: Breast parenchymal patterns and their changes with age. Br. J. Radiol..

[14] Maskarinec G., Pagano I., Lurie G., Wilkens L.R., Kolonel L.N. (2005). Mammographic density and breast cancer risk: The multiethnic cohort study. Am. J. Epidemiol..

[15] Sawada T., Akashi S., Nakamura S., Kuwayama T., Enokido K., Yoshida M., Hashimoto R., Ide T., Masuda H., Taruno K. (2017). Digital volumetric measurement of mammographic density and the risk of overlooking cancer in Japanese women. Breast Cancer.

[16] Nishiyama K., Taira N., Mizoo T., Kochi M., Ikeda H., Iwamoto T., Shien T., Doihara H., Ishihara S., Kawai H. (2020). Influence of breast density on breast cancer risk: A case control study in Japanese women. Breast Cancer.

[17] Sung H., Rosenberg P.S., Chen W.Q., Hartman M., Lim W.Y., Chia K.S., Yang X.R. (2015). Female breast cancer incidence among Asian and Western populations: More similar than expected. J. Natl. Cancer Inst..

[18] American College of Radiology (2016). Handlungsempfehlungen und Monitoring. ACR bi-RADS^®^-Atlas der Mammadiagnostik Richtlinien zu Befundung.

[19] Sprague B.L., Conant E.F., Onega T., Garcia M.P., Beaber E.F., Herschorn S.D., Lehman C.D., Tosteson A.N., Lacson R., Schnall M.D. (2016). Variation in mammographic breast density assessments among radiologists in clinical practice: A multicenter observational study. Ann. Intern. Med..

[20] Melnikow J., Fenton J.J., Whitlock E.P., Miglioretti D.L., Weyrich M.S., Thompson J.H., Shah K. (2016). Supplemental screening for breast cancer in women with dense breasts: A systematic review for the U.S. Preventive Services Task Force. Ann. Intern. Med..

[21] Gweon H.M., Youk J.H., Kim J.A., Son E.J. (2013). Radiologist assessment of breast density by BI-RADS categories versus fully automated volumetric assessment. AJR Am. J. Roentgenol..

[22] Youk J.H., Gweon H.M., Son E.J., Kim J.A. (2016). Automated volumetric breast density measurements in the era of the BI-RADS Fifth Edition: A Comparison with Visual Assessment. AJR Am. J. Roentgenol..

[23] Ekpo E.U., McEntee M.F. (2014). Measurement of breast density with digital breast tomosynthesis–a systematic review. Br. J. Radiol..

[24] Wanders J.O., Holland K., Veldhuis W.B., Mann R.M., Pijnappel R.M., Peeters P.H., van Gils C.H., Karssemeijer N. (2017). Volumetric breast density affects performance of digital screening mammography. Breast Cancer Res. Treat..

[25] Brand J.S., Czene K., Shepherd J.A., Leifland K., Heddson B., Sundbom A., Eriksson M., Li J., Humphreys K., Hall P. (2014). Automated measurement of volumetric mammographic density: A tool for widespread breast cancer risk assessment. Cancer Epidemiol. Biomark. Prev..

[26] Mandelson M.T., Oestreicher N., Porter P.L., White D., Finder C.A., Taplin S.H., White E. (2000). Breast density as a predictor of mammographic detection: Comparison of interval- and screen-detected cancers. J. Natl. Cancer Inst..

[27] Benson S.R., Blue J., Judd K., Harman J.E. (2004). Ultrasound is now better than mammography for the detection of invasive breast cancer. Am. J. Surg..

[28] Welch H.G., Prorok P.C., O’Malley A.J., Kramer B.S. (2016). Breast-cancer tumor size, overdiagnosis, and mammography screening effectiveness. N. Engl. J. Med..

[29] Vourtsis A., Berg W.A. (2019). Breast density implications and supplemental screening. Eur. Radiol..

[30] Berg W.A., Blume J.D., Cormack J.B., Mendelson E.B., Lehrer D., Böhm-Vélez M., Pisano E.D., Jong R.A., Evans W.P., Morton M.J. (2008). Combined screening with ultrasound and mammography vs mammography alone in women at elevated risk of breast cancer. JAMA.

[31] Ohuchi N., Suzuki A., Sobue T., Kawai M., Yamamoto S., Zheng Y.F., Shiono Y.N., Saito H., Kuriyama S., Tohno E. (2016). Sensitivity and specificity of mammography and adjunctive ultrasonography to screen for breast cancer in the Japan Strategic Anti-cancer Randomized Trial (J-START): A randomised controlled trial. Lancet.

[32] Harada-Shoji N., Suzuki A., Ishida T., Zheng Y.F., Narikawa-Shiono Y., Sato-Tadano A., Ohta R., Ohuchi N. (2021). Evaluation of adjunctive ultrasonography for breast cancer detection among women aged 40–49 years with varying breast density undergoing screening mammography: A secondary analysis of a randomized clinical trial. JAMA Net. Open.

[33] Wilczek B., Wilczek H.E., Rasouliyan L., Leifland K. (2016). Adding 3D automated breast ultrasound to mammography screening in women with heterogeneously and extremely dense breasts: Report from a hospital-based, high-volume, single-center breast cancer screening program. Eur. J. Radiol..

[34] Kelly K.M., Dean J., Comulada W.S., Lee S.J. (2010). Breast cancer detection using automated whole breast ultrasound and mammography in radiographically dense breasts. Eur. Radiol..

[35] van Zelst J.C., Tan T., Clauser P., Domingo A., Dorrius M.D., Drieling D., Golatta M., Gras F., de Jong M., Pijnappel R. (2018). Dedicated computer-aided detection software for automated 3D breast ultrasound; an efficient tool for the radiologist in supplemental screening of women with dense breasts. Eur. Radiol..

[36] Comstock C.E., Gatsonis C., Newstead G.M., Snyder B.S., Gareen I.F., Bergin J.T., Rahbar H., Sung J.S., Jacobs C., Harvey J.A. (2020). Comparison of abbreviated breast MRI vs digital breast tomosynthesis for breast cancer detection among women with dense breasts undergoing screening. JAMA.

[37] Gatta G., Cappabianca S., La Forgia D., Massafra R., Fanizzi A., Cuccurullo V., Brunese S., Tagliafico A., Grassi R. (2021). Second-Generation 3D Automated Breast Ultrasonography (Prone ABUS) for Dense Breast Cancer Screening Integrated to Mammography: Effectiveness, Performance and Detection Rates. J. Pers. Med..

[38] Kim S.H., Kim H.H., Moon W.K. (2020). Automated breast ultrasound screening for dense breasts. Korean J. Radiol..

[39] Massafra R., Bove S., Lorusso V., Biafora A., Comes M.C., Didonna V., La Forgia D. (2021). Radiomic Feature Reduction Approach to Predict Breast Cancer by Contrast-Enhanced Spectral Mammography Images. Diagnostics.

[40] Bakker M.F., de Lange S.V., Pijnappel R.M., Mann R.M., Peeters P., Monninkhof E.M., Emaus M.J., Loo C.E., Bisschops R., Lobbes M. (2019). DENSE Trial Study Group. Supplemental MRI Screening for Women with Extremely Dense Breast Tissue. N. Engl. J. Med..

[41] Kuhl C.K., Schrading S., Strobel K., Schild H.H., Hilgers R.D., Bieling H.B. (2014). Abbreviated breast magnetic resonance imaging (MRI): First postcontrast subtracted images and maximum-intensity projection-a novel approach to breast cancer screening with MRI. J. Clin. Oncol..

[42] Friedewald S.M., Rafferty E.A., Rose S.L., Durand M.A., Plecha D.M., Greenberg J.S., Hayes M.K., Copit D.S., Carlson K.L., Cink T.M. (2014). Breast cancer screening using tomosynthesis in combination with digital mammography. JAMA.

[43] Skaane P., Bandos A.I., Gullien R., Eben E.B., Ekseth U., Haakenaasen U., Izadi M., Jebsen I.N., Jahr G., Krager M. (2013). Comparison of digital mammography alone and digital mammography plus tomosynthesis in a population-based screening program. Radiology.

[44] Ciatto S., Houssami N., Bernardi D., Caumo F., Pellegrini M., Brunelli S., Tuttobene P., Bricolo P., Fantò C., Valentini M. (2013). Integration of 3D digital mammography with tomosynthesis for population breast-cancer screening (STORM): A prospective comparison study. Lancet Oncol..

[45] Lowry K.P., Coley R.Y., Miglioretti D.L., Kerlikowske K., Henderson L.M., Onega T., Sprague B.L., Lee J.M., Herschorn S., Tosteson A.N. (2020). Screening performance of digital breast tomosynthesis vs digital mammography in community practice by patient age, screening round, and breast density. JAMA Net. Open.

[46] Tollens F., Baltzer P.A.T., Dietzel M., Rübenthaler J., Froelich M.F., Kaiser C.G. (2021). Cost-Effectiveness of Digital Breast Tomosynthesis vs. abbreviated Breast MRI for Screening Women with Intermediate Risk of Breast Cancer-How Low-Cost Must MRI Be?. Cancers.

[47] Gardezi S.J.S., Elazab A., Lei B., Wang T. (2019). Breast cancer detection and diagnosis using mammographic data: Systematic review. J. Med. Internet. Res..

[48] Yi A., Chang J.M., Shin S.U., Chu A.J., Cho N., Noh D.Y., Moon W.K. (2019). Detection of noncalcified breast cancer in patients with extremely dense breasts using digital breast tomosynthesis compared with full-field digital mammography. Br. J. Radiol..

